# Cross-sectional study of antibiotic resistance to *Escherichia coli* and *Salmonella* spp. in cats in Yogyakarta, Indonesia, and Dili, Timor-Leste

**DOI:** 10.14202/vetworld.2024.2347-2354

**Published:** 2024-10-27

**Authors:** Widagdo Sri Nugroho, Antonino Do Karmo, Gustaf Eifel Silalahi, Elphan Augusta Kajang, Putu Cri Devischa Gallantiswara

**Affiliations:** 1 Department of Veterinary Public Health, Faculty of Veterinary Medicine, Universitas Gadjah Mada, Yogyakarta, Indonesia; 2 Technical Superior, Ministry of Agriculture and Fisheries, Timor-Leste;

**Keywords:** antibiotic resistance, cats, Dili, *Escherichia coli*, *Salmonella* spp. Yogyakarta

## Abstract

**Background and Aim:**

Antibiotics are used in veterinary clinics and animal hospitals to treat infectious diseases. However, the improper use of antibiotics causes antibiotic resistance, which threatens future disease therapeutics in pet animals. This study aimed to estimate the prevalence of *Escherichia coli* and *Salmonella* spp. in cats and their resistance to antibiotics in Yogyakarta Province, Indonesia (IDN), and Dili, Timor-Leste (TL).

**Materials and Methods:**

A total of 255 cat’s rectal swab samples from veterinary clinics and hospitals in Yogyakarta Province, IDN, and Dili, TL were collected. All samples were transferred aseptically into an enrichment medium and subjected to various culture tests for *E. coli* and *Salmonella* spp. identification. All identified isolates were tested for antibiotic sensitivity using Kirby−Bauer disk diffusion method.

**Results:**

This study successfully isolated *E. coli* from 172/255 (67.45%) rectal swab samples, that is, 122/188 samples (64.89%) from Yogyakarta Province, IDN, and 50/67 samples (74.6%) from Dili, TL. *Salmonella* spp. was isolated from 13/188 samples (6.91%) from Yogyakarta, IDN. The antibiotic susceptibility test indicated that more than 30% of *E. coli* were resistant to ampicillin (AMP) (IDN = 39.3%, TL = 50%) and tetracycline (TE) (IDN = 41.8%, TL = 42%), and more than 40% of *Salmonella* spp. were resistant to enrofloxacin (44%), TE (56%), streptomycin (61%), and AMP (83%).

**Conclusion:**

*E. coli* and *Salmonella* spp. succeeded isolation in cats from IDN and TL, and some isolates were resistant to antibiotics. Cats with diarrhea or digestive problems have a 9.5-fold increased risk of infection by *Salmonella* spp. Considering the prevalence of resistance to *E. coli* and *Salmonella* spp., it is important to manage antibiotic resistance distribution across companion animals and humans because both species share the same living environment.

## Introduction

Digestive problems are common in companion animals [1]. Most digestive problems in cats (82.59%) are caused by infectious factors, which are driven by pathogenic organisms, including bacteria (49.30%), viruses (37.57%), and protozoa (13.13%) [2–4]. Enterobacteriaceae, such as *Escherichia coli*, *Klebsiella* spp., and *Salmonella* spp., are organisms that can be found in the digestive tract of humans [5, 6]. Studies have shown that isolates of *Staphylococcus intermedius*, *E. coli*, and other bacteria from pet animals have developed resistance to a variety of antimicrobial agents, including species with a potential for zoonotic diseases and resistance phenotypes of clinical interest, such as methicillin-resistant *Staphylococcus aureus*, vancomycin-resistant enterococci, and multidrug-resistant *Salmonella* Typhimurium [7, 8].

Antibiotics are often used in treatment plans at veterinary clinics and hospitals. However, the inappropriate use of antibiotics leads to another problem, namely, antibiotic resistance. Antibiotic resistance is a complex problem involving multiple bacterial species that are affected by reservoirs, transfer routes, and resistance mechanisms, and it leads to therapeutic failure [7, 9]. Cases of *E. coli* and *Salmonella* spp. that express resistance are common in cats in many countries. A previous study in China [10] showed that the prevalence of *Salmonella* was 9.47% in dogs and 1.77% in cats. Another study demonstrated that clinical *E. coli* isolates collected from dogs and cats express resistance to at least one drug and resistance to commonly used first-tier beta-lactam antimicrobials [9]. Due to the widespread use of broad-spectrum antibiotics and their frequent contact with humans, pet animals are considered potential reservoirs for the transmission of antimicrobial resistance (AMR) to humans [11–13].

This study aimed to estimate the prevalence of *E. coli* and *Salmonella* spp. in cats and their resistance to commonly used antibiotics in Yogyakarta Province, Indonesia (IDN), and Dili, Timor-Leste (TL).Comparing these two areas will provide more evidence of the occurrence of antibiotic-resistant bacteria in both cities.

## Materials and Methods

### Ethical approval and informed consent

This study was approved by the Faculty of Veterinary Medicine, Universitas Gadjah Mada Ethical Commission No: 064/EC-FKH/Int./2022, and the cat owners agreed to participate and provided a verbal consent before collection of the samples.

### Study period and location

This study was conducted from August to December 2022. Rectal swab samples from cats were collected from clinics and veterinary hospitals in Yogyakarta Province, IDN, and Dili City, TL.

### Sample collection

A cross-sectional study was conducted. A questionnaire was provided to record information about their breed, age, and health condition. The sample was obtained by inserting a sterile swab into the rectum, which was immediately placed in a sterile tube containing 8 mL of rappaport vassiliadis (RVS) transport medium (Oxoid). The samples were stored in a cooler box at approximately 4°C and transported to the laboratory for further analysis. Rectal swab samples from Yogyakarta Province, IDN were sent to the Veterinary Public Health Laboratory of the Faculty of Veterinary Medicine, Universitas Gadjah Mada, IDN. Meanwhile, samples from Dili, TL, were sent to the Menzies School of Health Research laboratory in TL for bacterial isolation and identification.

### Bacterial isolation and identification

**Figure-1:**
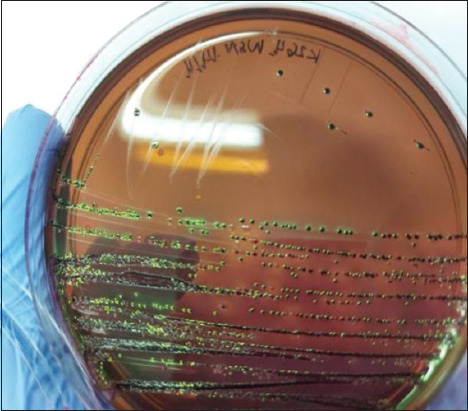
Appearance of *Escherichia coli* colonies grown on eosin methylene blue agar with black and metallic sheen.

**Figure-2:**
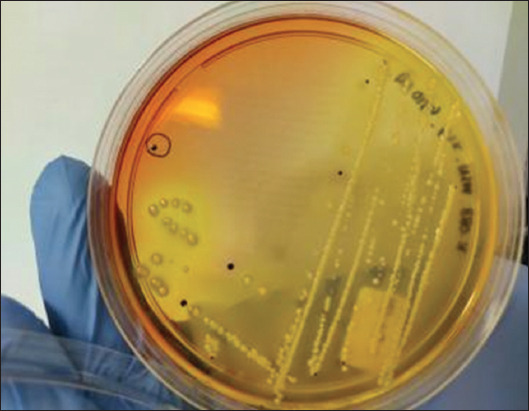
Sample of *Salmonella spp*. colonies grown on xylose lysine deoxycholate agar (circle line: Central black).

A loopful of the RVS sample solution was taken and streaked onto eosin methylene blue selective agar medium (Oxoid) without antibiotics, followed by incubation at 37°C for 18–24 h. *E. coli* colonies appeared as a metallic green sheen (Figure-1). Presumptive *E. coli* colonies were selected from each pure isolate plate for further biochemical identification using triple sugar iron agar and lysine iron agar media after incubation at 37°C for 18–24 h [14, 15]. One milliliter of the RVS solution was cultured in 9 mL of tetrathionate broth (TTB) solution (Oxoid) at 37°C for 18–24 h to isolate *Salmonella* spp. A loopful of the TTB sample solution was then streaked onto xylose lysine deoxycholate agar and incubated at 37°C for 18–24 h. Suspected *Salmonella* spp. colonies were identified by their red color with black centers on the plate (Figure-2). These colonies were selected for subsequent subculturing to obtain pure isolates [16].

### Antibiotic susceptibility test

**Figure-3:**
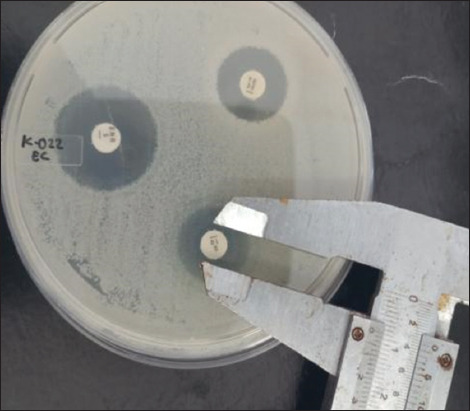
Result of sensitivity test of *Escherichia coli* isolate (sample no K.187) in Mueller Hinton agar; clear zones were observed for tetracycline, streptomycin, and enrofloxacin.

**Figure-4:**
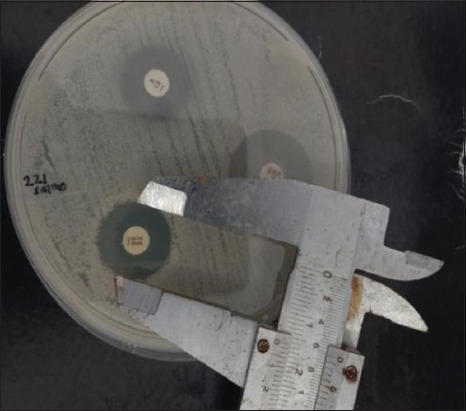
Result of sensitivity test of *Salmonella* spp. isolate (sample no. K.008) in Mueller Hinton agar; clear zones were observed around tetracycline, enrofloxacin, and streptomycin.

Antibiotic susceptibility was assessed using the Kirby−Bauer disk diffusion method with four antibiotic agents mostly used in companion animals, including ampicillin (AMP, 10 μg), enrofloxacin (ENR, 5 μg), tetracycline (TE, 30 μg), and streptomycin (S, 10 μg) (Oxoid). All antibiotic concentrations and resistance were determined according to the inhibition zone diameter on the agar Mueller Hinton agar plate (Figures-3 and 4) and were compared with those of the Clinical Laboratory Standards Institute [17].

### Statistical analysis

Data on breed, age, clinical status, bacteriology identification, and antibiotic susceptibility were analyzed descriptively. The association between bacterial infection and risk factors was analyzed using the Chi-square test and odds ratio (OR) to measure the association’s strength (p < 0.05). Data management and analysis were performed using the Statistical Package for the Social Sciences statistical software Version 28 (IBM Corp., New York, USA).

## Results

In total, 255 cat rectal swab samples were collected from clinics and animal hospitals in Yogyakarta province, IDN (188 cats), and Dili, TL (67 cats). Rectal swabs were obtained from six regions from both IDN and TL; 108 cats from Sleman district (42.3%), 48 cats from Yogyakarta city (18.8%), 15 cats from Bantul district (5.8%), 9 cats from Gunung Kidul district (3.5%), 8 cats from Kulon Progo district (3.1%), and 67 cats from Dili (26.2%). Cats comprised 148 (58%) males and 107 (42%) females; 89 (35%) kittens (<1 years), 140 (55%) juniors (1–5 years), and 26 (10%) seniors (>5 years). This research successfully isolated *E. coli* from a total of 172–255 samples (67.45%), that is, 122–188 samples (64.89%) from Yogyakarta Province, IDN, and 50–67 samples (74.6%) from Dili, TL. *Salmonella* spp. were isolated from 13 samples (6.91%) from DIY, but no samples were positive for Dili and TL.

Regarding the cats’ dietary and drinking behaviors, of the 255 analyzed cats, 198 cats were fed using commercial pet foods (78%), 12 cats were fed using home-prepared food (5%), and the remaining were fed a combination of both food types (17%). The majority of cats drank mineral water (65%), 29% drank raw water, and only 4% drank both mineral and raw water. Vaccines and deworming are critical components in the management of both infectious and non-communicable diseases in pet animals. In this study, as many as 107 (42%) and 139 (55%) cats were vaccinated and not vaccinated, respectively. Deworming history also showed that 138 cats had regular deworming for <3 months (54%), 24 cats had regular deworming for more than 3 months (9%), 74 cats never had deworming (29%), and 19 cats had an unknown deworming history (7%).

Based on statistical analysis, this study showed no association (p > 0.05) between the variables related to cat conditions (breed, age, sex, diet, and source of drinking water) and medical history (vaccination status, deworming history, and medicated history) (Table-1). However, diarrhea was identified as a variable associated with the presence of *Salmonella* spp. in cats’ rectal swab samples (p < 0.05, OR: 9.25, confidence interval: 1.56–11.05).

**Table-1:** Frequency of isolation of *Escherichia coli* and *Salmonella* spp. from cats in Yogyakarta province, Indonesia, and Dili, Timor-Leste.

Variables	Categories	No. of cat samples	*Escherichia coli*			*Salmonella* spp.		
	
			No. of positive (%)	Chi- square	p-value	No. of positive (%)	Chi- square	p-value
Sub-Regency location	Sleman	108	74 (68.5)	13.02	0.23	12 (11.1)	8.95	0.11
	Yogyakarta City	48	28 (58.3)			4 (8.3)		
	Bantul	15	6 (40)			1 (6.7)		
	Gunung Kidul	9	9 (100)			0 (0)		
	Kulon Progo	8	5 (62.5)			1 (12.5)		
	Dili	67	50 (74.6)			0 (0)		
Breed	Domestics	141	102 (72.3)	3.57	0.16	8 (5.6)	1.02	0.39
	Pedigree	52	31 (59.6)			5 (9.6)		
	Mix breed	62	39 (62.9)			5 (8)		
Age	Kitten (<1 years)	89	66 (74.1)	3.18	0.36	9 (10.1)	0.19	0.66
	Junior (1–5 years)	140	90 (64.2)			9 (6.4)		
	Senior (>5 years)	26	16 (61.5)			0 (0)		
Sex	Males	148	93 (62.8)	3.41	0.64	11 (7.4)	0.07	0.78
	Females	107	79 (73.8)			7 (6.5)		
Diet	Home-prepared	12	8 (66.6)	2.15	0.34	4 (33.3)	1.53	0.21
	Commercial	198	129 (65.1)			12 (6)		
	Combination feed	43	33 (76.7)			2 (4.6)		
Source of drinking water	Raw water	75	55 (73.3)	1.82	0.4	5 (6.6)	0.97	0.61
	Mineral water still exists	167	108 (64.6)			13 (7.7)		
	Combination water	11	7 (63.6)			0 (0)		
Clinical status	Diarrhea	55	37 (67.2)	0.75	0.68	9 (16.3)	9.25	0.002*
	Non-diarrhea	190	127 (66.8)			7 (3.7)		
	Unknown	10	8 (80)			2 (20)		
Vaccination status	Vaccinated	107	67 (62.6)	5.64	0.06	9 (8.4)	1.05	0.59
	Not vaccinated	139	96 (69)			9 (6.4)		
	Unknown	9	9 (100)			0 (0)		
Deworming history	<3 months	138	89 (64.4)	1.58	0.66	8 (5.7)	6.32	0.97
	>3 months	24	18 (75)			1 (4.1)		
	Never given	74	51 (68.9)			5 (6.7)		
	Unknown	19	14 (73.6)			4 (21)		
Medicated history	Yes	130	85 (65.3)	0.30	0.22	9 (6.9)	0.73	0.96
	No	114	77 (67.5)			8 (7)		
	Unknown	11	10 (90.9)			1 (9)		

Some antibiotics, such as amoxicillin, AMP, penicillin, S, ENR, TE, and cephalosporin, were the first choice to treat the pet [18, 19]. These antibiotics are commonly used to treat pet animals in Yogyakarta, IDN, and Dili, TL. This study focuses on investigating the susceptibility of *E. coli* and *Salmonella* spp. to AMP, ENR, S, and TE.

The antibiotic susceptibility of *E. coli* isolates is shown in Table-2 [17]. The highest rate of resistance to IDN isolates was found in 51 of 122 *E. coli* isolates resistant to TE (41.8%), followed by AMP (39.3%), S (27.0%), and ENR (13.9%). However, >50% of the isolates were still sensitive to AMP (61/122), ENR (65/122), and S (60/122). Compared with the TL *E. coli* isolates, the highest resistance was found in 25 of 50 isolates to AMP (50%), followed by TE (42%), S (38%), and ENR (2%). Twelve *E. coli* isolates were identified as multiple drugs resistant to four antibiotics (AMP, ENR, TE, and S). One isolate was from Dili, and the others were from IDN.

**Table-2:** Antimicrobial susceptibility of *Escherichia coli* isolates from cats in Yogyakarta province and Dili.

Antimicrobial agent	CLSI, 2020 range (mm) [17]			AMR (%)			AMR (%)			Total
	
				Yogyakarta, Indonesia (n = 122)			Dili, Timor-Leste (n = 50)			
			
	S	I	R	S	I	R	S	I	R	S
Ampicillin	≥17	14–16	≤13	61 (50.0)	13 (10.6)	48 (39.3)	15 (30)	10 (20)	25 (50)	73
Enrofloxacin	≥23	17–22	≤16	65 (53.2)	40 (32.7)	17 (13.9)	42 (84)	7 (14)	1 (2)	18
Tetracycline	≥15	12–14	≤11	54 (44.2)	17 (13.9)	51 (41.8)	27 (54)	2 (4)	21 (42)	72
Streptomycin	≥15	12–14	≤11	60 (49.1)	29 (23.7)	33 (27.0)	22 (44)	9 (18)	19 (38)	52

S=Susceptible, I=Intermediate, R=Resistance, AMR=Antimicrobial resistance

The antibiotic sensitivity test results for 18 IDN-infected cats were positive for *Salmonella* spp. (Table-3) [17]. In the present study, resistance to AMP was the most prevalent among 15 isolates (83%), followed by resistance to S (61%), TE (56%), and ENR (44%). Seven isolates were recognized as multidrug-resistant to four antibiotics.

**Table-3:** Antimicrobial susceptibility of 18 *Salmonella* spp. isolates from cats in Yogyakarta province.

Antimicrobial agent	CLSI, 2020 range (mm) [17]			No. of isolates		
	
	S	I	R	S (%)	I (%)	R (%)
Ampicillin	≥17	14–16	≤67	3 (17)	0 (0)	15 (83)
Enrofloxacin	≥23	17–22	≤23	5 (28)	5 (28)	8 (44)
Tetracycline	≥15	12–14	≤45	7 (39)	1 (6)	10 (56)
Streptomycin	≥15	12–14	≤45	6 (33)	1 (6)	11 (61)

S=Susceptible, I=Intermediate, R=Resistance

## Discussion

In this study, of the 255 cats collected, 55 cats (21.5%) had diarrhea, 190 cats (74.5%) had no history of diarrhea, and 10 cats (3.9%) had an unknown history in the past 2 weeks during screening in the clinics. Out of 255 cats, 45 showed symptoms of diarrhea, and 15 exhibited a decrease in appetite. Unfortunately, no scoring system was used to assess the severity or type of diarrhea condition. Diarrhea was a significant variable associated with the presence of *Salmonella* spp. in cats but not in *E. coli*. The presence of *Salmonella* spp. in the rectum was 4.15 times higher in cats with diarrhea than in those with no signs or history of diarrhea. Diarrhea is a clinical syndrome associated with salmonellosis in cats [20].

The antibiotic susceptibility of *E. coli* isolates showed that >30% were resistant to a group of β-lactam antibiotics (AMP) and TE. The highest resistance to IDN samples was observed for 51 *E. coli* isolates to TE (41.88%) compared with TL isolates and was highest for 25 isolates to AMP. Recent research in Europe also found that the most common resistances to *E. coli* were AMP (18%), sulfamethoxazole (15%), and TE (14%) [11]. However, more than 50% of IDN isolate susceptibility was still sensitive to AMP (61/122) and ENR (65/122) compared with 50% of TL isolate susceptibility was still sensitive to ENR (42/50) and TE (27/50).

*E. coli*, *Klebsiella* spp., *Salmonella* spp., and *Enterobacter* spp. are members of *the Enterobacteriaceae* family. Isolation results identified *E. coli* in 172/255 cats and *Salmonella* spp. in 18/255 cats [5, 6]. Many organisms belonging to these species are symbiotic with the gastrointestinal tract, and an increase in antibiotic resistance in this family is envisioned to cause problems in public health [21, 22]. Its typical natural and capacity to serve as a reservoir for antibiotic resistance genes that may be transmitted to other pathogens by horizontal gene transfer indicates that the level of resistance in Enterobacteriaceae is a potential marker of AMR in the bacterial pathogens of cats and other companion animals [23–25].

According to Morato *et al*. [26], enteropathogenic (EPEC) *E. coli* was found in 14 of 300 domestic cats with diarrhea and non-diarrhea domestic cats, from São Paulo, Brazil. The identified EPEC strains were heterogeneous, including those that infect humans. Some strains of *E. coli* present in the companion animal’s digestive system were also resistant to several types of antibiotics [6, 27]. Some of these strains are also found infecting humans, including NDM-1-producing *E. coli* in USA, OXA-48-producing *E. coli* in Europe, NDM-5 *E. coli* from dog ear in Finland, and CTX-M-15-producing *E. coli* in China [6]. The discovery of these extended beta-lactamase (ESBL) enzymes in *E. coli* should be considered because they are widely spread among environments, humans, and animals [28]. CTX-M-producing *E. coli* was found in various types of environments, such as rivers, wastewater, drinking water, soil, and vegetables. In addition, the bacteria have been detected in several animal species, including livestock, companion animals, and even wildlife animals [28–30]. *E. coli* is one of the main bacteria reported to produce OXA and NDM enzymes, which are mostly found in cattle, wildlife, and companion animals, suggesting cross-species transmission [31]. ESBL-producing *E. coli* transmission between humans and animals living in the same house has been studied in several studies [32, 33]. A Finnish study captured the transmission of ST167 NDM-5 and ST69 CTX-M *E. coli* between two dogs and humans living together. However, in this study, the authors considered that NDM-5 was likely transmitted from humans to dogs [33].

*Salmonella* serotypes can be categorized into host-restricted, host-specific, and generalist types, each with significant implications for epidemiology and public health [34]. A recent study estimated that approximately 93.8 million human cases of gastroenteritis and deaths occur due to *Salmonella* infection around the world each year. Salmonellosis is primarily recognized as a foodborne illness, and it is estimated that approximately 9% of cases result from direct contact with animals [16, 34].

Based on the diarrhea status, 18 samples were positive for *Salmonella* spp., 9 cats with diarrhea 16.3% (9/55), 7 cats without diarrhea 3.7% (7/190), and 2 cats had an unknown status of 20% (2/10). The findings indicated that diarrhea is a significant clinical syndrome of *Salmonella* spp. infection in cats. This study showed that *Salmonella* spp. was 9.25 times more frequently found in cats with diarrhea than those without diarrhea symptoms. According to Arsevska *et al*. [35], dogs and cats generally exhibit subclinical *Salmonella* infection.

*Salmonella* spp. was also found in healthy dogs, with a prevalence of 1.85% among 325 dogs [36]. Another study from the USA that collected dog and cat feces for around 2 years reported that the prevalence of *Salmonella* spp. in diarrheic dogs was 3.8% and that in non-diarrheic dogs was 1.8%. Three of the 542 cats (<1%) were positive for *Salmonella* spp., with one being a non-diarrheic cat, and two being diarrheic cats [37]. *Salmonella* is an important foodborne pathogen worldwide.

Eighteen *Salmonella* spp. isolates were found in IDN. The results of the antibiotic susceptibility test showed resistance to AMP, which was the most prevalent among 15 isolates (83%), followed by resistance to S (61%), TE (56%), and ENR (44%). In Xuzhou, Jiangsu Province, China, the resistance rate of the *Salmonella* strain from cats and dogs to TE was the most prevalent among 25 strains (92%); among the 23 multidrug-resistant isolates, resistance to TE, azithromycin, and cefazolin was most often observed [10]. In Taiwan [38], the resistance rates of *Salmonella* spp. strains from dogs to sulfamethoxazole/trimethoprim (37.5%) and TE (77.5%) were similar to those observed in the present study, but resistance to other antibiotic agents, such as AMP, was lower than that in the present study.

The presence of multidrug-resistant *Salmonella* spp. in animals, humans, and the environment was captured in a study conducted in South Africa [39]. Most of the bacterial isolates were resistant to sulfonamide, a combination of ENR and erythromycin, oxytetracycline, imipenem, TE, and trimethoprim. Although this study did not directly illustrate bacterial transmission among animals, humans, and animals, its occurrence has been an indicator of cross-contamination of AMR distribution.

The presence of multidrug-resistant *E. coli* and *Salmonella* spp. in IDN and TL cats should be an important concern because these bacteria are ubiquitous in the environment. Moreover, the bacteria were also found in non-diarrheic or healthy cats. Transmission from cats to humans is strongly possible. Some studies have reported that contact with pets and having pets in homes increase ESBL-producing *E. coli* carriage rates in humans [40, 41]. According to Meyer *et al*. [40], the rates of human contact with pets are higher. Even so, there are not many studies demonstrating the transmission of *Salmonella* spp. directly from cats to humans; however, finding the human-related serovar of *Salmonella* spp. in cats, such as *S*. Typhimurium and *S. enteritidis*, can also be an indication of transmission [10, 34, 42]. Our study indicates that cats with diarrhea or digestive problems and those who are asymptomatic or appear healthy may be carriers of *Enterobacteriaceae* that are resistant to the antibiotic agents tested. They can be a potential source of human infection. Moreover, it is also emphasized that both humans and animals receive treatment with the same antibiotic agents.

## Conclusion

In Yogyakarta Province, IDN, the prevalences of *E. coli* and *Salmonella* spp. in cats were 64.89% and 6.91%, respectively. In Dili, TL, 74.6% of the cats tested positive for *E. coli*, but no *Salmonella* spp. was found. The highest resistance was observed in IDN samples, with 41.88% of the 51 isolates resistant to TE, whereas TL isolates exhibited the highest resistance to AMP among 25 isolates. Twelve *E. coli* isolates were identified as multi drug resistant to 4 antibiotics (AMP, ENR, TE, and S). *Salmonella* spp. isolates exhibited the highest resistance to AMP (83%), followed by S (61%), TE (56%), and ENR (44%). Seven isolates were multidrug-resistant to all four antibiotics.

Clinically, healthy cats may act as carriers of antibiotic-resistant *Enterobacteriaceae*, with some isolates showing multidrug resistance, particularly to antibiotics commonly used in human medicine.

## Authors’ Contributions

WSN: Conceptualized and designed the study, organized the research activity, and reviewed the manuscript. ADK: Designed the study, data collection and analysis in TL, and reviewed the manuscript. GES: Designed the study, collected and analyzed data, and drafted the manuscript. EAK: Sample collection and laboratory analysis. PCDG: Data analysis and drafted and revised the manuscript. All authors have read and approved the final manuscript.
